# Impact of polyacrylic acid as soil amendment on soil microbial activity under different moisture regimes

**DOI:** 10.1038/s41598-025-04457-8

**Published:** 2025-06-03

**Authors:** Christian Buchmann, Simon Rudolph, Janina Neff, Zacharias Steinmetz

**Affiliations:** IES Landau, Institute for Environmental Sciences, Group of Soil and Environmental Chemistry, RPTU University Kaiserslautern-Landau, Landau, Germany

**Keywords:** Polyacrylic acid, Soil amendment, Soil microbial activity, Substrate-induced respiration, Soil water holding capacity, Drying-rewetting cycles, Microbiology, Biogeochemistry, Environmental sciences, Natural hazards, Solid Earth sciences, Materials science

## Abstract

Polyacrylic acid (PAA), a synthetic superabsorbent polymer (SAP), enhances the maximum water holding capacity (WHC_max_), soil structural stability, and aeration of soil but may simultaneously affect soil microbiome by altering soil properties. However, its effects on microbial activity under different moisture regimes remain insufficiently understood. We examined the impact of PAA on soil microbial activity in a sand and loam treated with PAA at three concentrations (25, 250, 2500 mg kg^− 1^ dry soil) either incubated under constant moisture or subjected to ten drying-rewetting cycles. During incubation, soil WHC_max_, pH, and soil microbial activity via headspace CO_2_ and MicroResp assay were measured. PAA increased WHC_max_ in both soils, yet its effectiveness decreased in loam under static conditions. Initially, PAA acidified both soils, with pH shifts persisting in sand but dissipating in loam after one week. Drying–rewetting cycles counteracted acidification and partially maintained PAA swelling. In sand, high PAA concentrations consistently suppressed microbial respiration across substrate groups, whereas in loam, microbial responses were modulated by moisture dynamics: drying–rewetting enhanced, while static moisture reduced SIR under PAA treatment. Overall, PAA altered soil microbial activity in a concentration-, soil type-, and moisture-dependent manner, emphasizing the dual role of SAPs in improving soil water retention while potentially impairing microbial-mediated soil functions over time.

**Supplementary Information:**

The online version contains supplementary material available at 10.1038/s41598-025-04457-8.

## Introduction

Superabsorbent polymers (SAPs) can absorb and retain large amounts of water or aqueous solutions, which makes them commonly used as soil conditioner to alter soil properties, such as the maximum water holding capacity (WHC_max_), soil structural stability, as well as the availability of nutrients, fertilizers, and pesticides^1,2^. Based on their properties and synthesis methods, SAPs can be categorized by their origin (natural, synthetic, or hybrid) and crosslinking type (chemical or physical bonds)^3^. In this context, polyacrylic acid (PAA) is one of the most frequently used synthetic SAPs due to its high water absorption (swelling) capacity, typically ranging between 300 and 500 mL water g^− 1^ dry polymer^4^. However, the water absorption capacity of SAPs can be significantly impaired by the presence of certain ions, particularly multivalent cations (e.g., Ca^2+^, Al^2+^), through cation-mediated crosslinking^5^. This process occurs when multivalent cations interact with anionic groups on the polymer chains, forming additional crosslinks within the network structure and resulting in reduced water absorption capacity and altered key physicochemical properties, including mechanical strength, diffusion rates, and pH sensitivity^6–8^.

While the beneficial properties of superabsorbent polymers (SAPs) are well-documented, their introduction into soil systems may pose several risks and challenges. A major concern is the persistence of PAA due to its extremely low biodegradation rates of only 0.2–0.5% y^− 1^ in soil^9^. In contrast to naturally occurring SAPs such as gellan, alginate, or mucilage, this persistence raises the potential for long-term accumulation, with reported concentrations reaching up to 10 g SAP kg^− 1^ soil^6^. Moreover, it has been shown that the positive effects of SAPs on soil WHC can diminish within three months after application, necessitating frequent reapplications to maintain effectiveness in arid and semi-arid soils and thereby further increasing concerns about the accumulation and impact of PAA in soil^10^. Additionally, soil moisture dynamics, such as regular drying-rewetting cycles, can significantly alter the functionality of SAPs, e.g., leading to reduced water absorption capacity over time^11^. Aging processes, including chemical, photolytic, and mechanical transformation and degradation, might further contribute to the potential fragmentation of PAA into smaller particles. These fragments can get into deeper soil layers or into neighboring ecosystems with potential negative effects on the environment^12^.

While PAA is well-recognized for its beneficial impacts on various soil physicochemical properties, the consequences of its persistence, accumulation, and aging on soil microbial activity remain largely unknown^6^. Given the essential role of soil microbial activity in nutrient cycling, organic matter (OM) decomposition, and overall soil health^13,14^, it is crucial to consider how synthetic SAPs such as PAA might affect, e.g., soil microbial diversity, metabolic functioning, and respiration dynamics to better understand the long-term implications on soil ecosystems. On the one hand, PAA may disrupt or alter microbial processes by altering soil structure and porosity^15,16^, which potentially affects the availability of microbial habitats, water distribution, and oxygen diffusion throughout the soil. On the other hand, the chemical properties of PAA, particularly its capability to form complexes with essential nutrients, its presence as a carbon source, and its potential influence on soil pH might also affect the microbial growth and their metabolic activity^17,18^. Thus, its persistence and accumulation potential could make PAA a long-term stressor to microbial communities, altering their functional capabilities and community dynamics through adaptive responses over time.

Soil microbial respiration can be divided into autotrophic respiration from plant roots and associated microorganisms using self-produced energy and heterotrophic respiration from soil microorganisms decomposing OM^19^. Since heterotrophic respiration is heavily influenced by environmental factors such as soil moisture, temperature, carbon availability, and pH^20,21^, any disturbances or amendments might lead to the alteration of microbial activity and soil respiration dynamics. Basal respiration, typically measured via CO_2_ release, primarily results from substrate availability in soil and provides fundamental information on microbial physiology and maintenance requirements. It thus serves as a measure of metabolic activity without quantifying the active microbial activity, as only the currently active microorganisms involved in respiration are recorded. In contrast, substrate-induced respiration (SIR) via the application of various substrates (e.g., glucose) can activate a broader spectrum of the soil microbiome, including previously dormant taxa^22,23^. This approach allows a more detailed assessment of microbial activity and functional diversity in soil with the quantity and range of utilized carbon sources reflecting microbial biomass abundance and community functional adaptability.

Despite the key role of microbial activity for diverse soil processes and soil properties, the specific interactions with and effects of PAA are subject to current research, especially with respect to soil moisture dynamics. This is mainly because most of the available studies are limited to the effect of SAPs on basic soil properties important for agriculture, like aggregate stability, porosity, soil organic matter (SOM) content, and nutrient availability^24^. Consequently, we aimed to gain first insights into the concentration-, time-, and moisture dynamic-dependent effects of PAA on soil microbial activity and soil functional diversity. For this, a 10-week incubation experiment was performed with two soils, a loam and a sand, which were both treated with PAA at three different concentrations (25, 250, and 2500 mg PAA kg^− 1^ dry soil) and subjected to either constant moisture or drying-rewetting conditions. Throughout the incubation, both substrate-induced and basal soil respiration were recorded using MicroResp and headspace CO_2_ measurements. In addition, the maximum water-holding capacity (WHC_max_) and the pH of the soils were monitored and changes in the soil structure were investigated using scanning electron microscopy (SEM).

We hypothesized that (1) PAA will initially increase the WHC_max_ of the PAA-treated soils in a concentration-dependent manner; however, this effect should diminish over time, particularly when subjected to moisture dynamics. Furthermore, we hypothesized that (2) PAA-induced shifts in soil pH and water binding modulate soil microbial activity, with low PAA concentrations enhancing microbial respiration via improved water availability, and high PAA concentrations suppressing it due to soil acidification and stronger water binding. As PAA alleviates drought stress, we expected it to mitigate negative effects of drying–rewetting cycles on soil microbial activity. Consequently, we assumed that (3) negative effects of PAA and moisture dynamics, both individually and in combination, are reflected in changed substrate usage patterns, in which simpler, more readily available carbon sources are preferred over complex compounds. However, we also hypothesized that (4) PAA loses its functionality over time due to aging processes - such as crosslinking induced by polyvalent cations and sorption to soil minerals and SOM - further accelerated by drying–rewetting cycles^6,25^. These processes reduce the water absorption capacity of interparticulate PAA hydrogel and lead to a more rapid attenuation of its effects on soil microbial respiration under cyclic compared to static moisture conditions.

## Materials and methods

### Soil samples and sample preparation

Two well-characterized reference soils (Lufa 2.1 and 2.4) from the Agricultural Analysis and Research Institute Speyer (LUFA, Speyer, Germany) were used in this study as typical agricultural soils. Their textures are classified as sand (Lufa 2.1) and loam (Lufa 2.4) with a pH of 4.6 and 7.5, and an organic carbon content (C_org_) of 0.6 and 1.8%, respectively. An overview of selected soil physicochemical properties is presented in (Table 1).

**Table 1:** Selected physicochemical properties of the two investigated soils.

Parameter	Lufa 2.1	Lufa 2.4
Type	sand	loam
Sand (%)	88.2	33.1
Silt (%)	8.4	42.2
Clay (%)	3.5	23.7
C_org_ (%)	0.6	1.8
N_tot_ (%)	0.06	0.23
pH (0.01 M CaCl_2_)	4.6	7.5
CEC (meq 100 g^−1^)	2.9	17.4
WHC_max_ (g 100 g^−1^)	30	47
Bulk density (g cm^−3^)	1.47	1.18

Prior to the first moistening, the 2 mm-sieved air-dried soils were oven-dried at 30 °C for 4 days, subsequently spiked with high-weighted, linear polyacrylic acid (PAA) powder in its acid form (CAS 9003-01-4; M_v_ = 4.000.000 g mol^− 1^; ~ 0.1% cross-linkage, pH = 2.5–3.5) (Sigma-Aldrich, Germany) at three concentrations (25, 250 and 2500 mg PAA kg^− 1^ dry soil), and manually homogenized for 10 min. Control soils without PAA were prepared accordingly. All treatments were prepared in triplicate by weighing 50.06 ± 0.08 g of the sand and 40.04 ± 0.05 g of the loam into sealable petri dishes (WHC_max_ and pH determination) and screw-cap jars (headspace CO_2_ measurements), respectively. For MicroResp measurements, 0.60 ± 0.01 g of the sand and 0.50 ± 0.01 g of the loam were weighed directly into deep-well plates.

The prepared samples were subdivided into two groups based on the soil moisture conditions during the incubation time (constant moisture and drying-rewetting conditions). Throughout the 10-week incubation, all samples were kept closed in the dark at constant 20 °C using a ventilated universal oven (Memmert, Schwabach, Germany), with one half subjected to ten drying–rewetting cycles and the other maintained at 40% WHC_max_ throughout the 10-week incubation. All ten drying–rewetting cycles involved (1) a rapid (re)wetting event with ultra-pure water, followed by (2) a three-day wet incubation period at 20 °C, and (3) a four-day drying phase at 30 °C until complete dryness. All incubation vessels provided sufficient headspace and thus eliminated the need for external aeration during the humid period, while gravimetric controls confirmed consistently stable water content throughout.

At the cycles 0, 3, 5, and 10, corresponding to the respective incubation weeks, soil replicates for each treatment and control were taken and investigated for physicochemical properties and soil microbial activity. To improve readability, the term ‘measurement point’ (MP) is used to include soil samples incubated under constant moisture conditions as well as cyclically dried and remoistened (e.g., measurement point 3 depicts incubation week 3 for the constant and the third drying-rewetting cycle for the cyclic soil samples, respectively).

Additionally, pure PAA hydrogel was prepared to evaluate its swelling potential in the different soil solutions and demineralized water by allowing dry PAA powder to form a completely swollen hydrogel for 48 h with an excess of soil water (or demineralized water), which was extracted from the respective soils for 30 min at 2900 rpm using a MegaStar 4.0 centrifuge (VWR, Germany) and 3D-printed polymer falcon tube centrifugation inserts^26^. After 48 h, excess solution was separated from the swollen PAA hydrogel by centrifugation at 1000 × *g* for 20 min and the hydrogels were weighed to determine the amount of adsorbed solution per g dry PAA powder according to Buchmann et al.^27^.

### Soil physicochemical characterization

WHC_max_ (mL 100 g^− 1^ dry soil) of the soil samples was determined gravimetrically using the funnel method^28^ based on the weight difference between the wet soil samples (S_W_ / 100% WHC_max_) after 4 h of saturation and 14 h of gravitational draining, and the corresponding dry soil samples (S_D_ / 0% WHC) after 4 days of drying at 105 °C (Eq. 1).

1 $$\:{WHC}_{max}=\frac{{S}_{W}-{S}_{D}}{{S}_{D}}*100$$

Soil pH was measured using a multimeter (Consort, Belgium) at the measurement points 0, 3, 5, and 10 following PAA addition for both soils to monitor changes in soil acidity under the different moisture regimes.

### Microbial activity

#### MicroResp

Soil microbial respiration, including both basal and substrate-induced respiration, was measured using the MicroResp method^29^. For each combination of soil type, PAA concentration, moisture condition, and measurement point, a 96-deepwell plate was prepared containing eight different carbon substrates for SIR measurements. The following substrates were selected based on their ecological relevance, their documented presence in soil environments (e.g., as components of plant root exudates), and their ability to represent a broad spectrum of structural complexity: Glucose (GLU), Galactose (GAL), L-alanine (ALA), N-acetylglucosamine (NAGA), α-cyclodextrin (ACYC), Trisodium citrate (CIT), ɣ-aminobutyric acid (GABA), and ultrapure water (WAT) as control. This resulted in 12 replicates per substrate and plate. For the analysis, the substrates were grouped into carbohydrates (GLU, GAL, ACYC), amines (ALA, NAGA, GABA), and carboxylic acids (CIT).

Before substrate addition, the soils were preincubated at 40% WHC_max_ for three days. Substrate application to each well resulted in the addition of 30 mg C ml^− 1^ soil water and raised the soil moisture to 60% WHC_max_. CO₂ emissions were measured after 6 h using a pH color reaction with cresol red as the indicator in the agar gel of the 96-well microplates that covered the deepwell plates during incubation. Light absorption at 572 nm, measured using a microplate spectrophotometer (Infinite M200, Tecan, Switzerland), was used to quantify the color change in the indicator gel due to the evolved CO_2_. Non-linear calibration data were used to convert the changes in light absorbance before and after the 6-hour incubation into respiration rates (µg CO_2_-C g^− 1^ soil h^− 1^). SIR was calculated as the difference between the basal respiration rate (WAT) and the respiration rates from the various carbon sources. SIR values for each substrate, along WAT, were summed to determine the total substrate-induced respiration (SIR_tot_). Additionally, the variation coefficient of the SIR values was calculated. The respiratory response to the substrate added reflects the proportion of active microbial biomass that is correspondingly able to use the respective carbon source.

#### Headspace CO_2_ measurements

Headspace CO_2_ measurements were conducted to obtain basal soil respiration by assessing the CO_2_ concentration evolved in the headspace of the soil samples placed in incubation jars. Measurements were conducted according to^30^ using a portable Los Gatos greenhouse gas analyzer (Los Gatos Research Inc, ABB Ltd, Switzerland) in a closed-loop setup. For this, the incubation jars were equipped with a Luer-lock connector, a three-way valve, an injection cap and two gaskets. This setup allowed headspace sampling without a permanent septum connection, reducing CO_2_ loss. A preliminary experiment determined the CO_2_ loss of the jars (*n* = 5; 30 ± 6 ppmv CO_2_ h^− 1^), which was used as correction factor in the main experiment.

The headspace measurements included three days of respiration, starting with each rewetting of a drying-rewetting cycle. Static samples were vented during the four-day drying period of the cyclic samples. Prior to the removal of the sampling volume (V_s_ = 500 µL), the headspace of the jars was homogenized by mixing the volume with a syringe attached to the three-way valve. The internal volume of the closed-loop setup (V_l_), and thus the dilution factor, was determined prior to each measurement point using triplicate injections of standard gas with a known CO_2_ concentration (20,000 ± 400 ppmv). The CO_2_ concentration of the samples (X_s_; CO_2_ in ppmv) was calculated according to Eq. 2 as the difference (ΔX) between the baseline gas concentration before the injection (X_0_) and the average equilibrium concentration (30 s) after injection (X_i_).

2 $$\:{X}_{s}=\frac{{V}_{l}}{{V}_{s}}\varDelta\:X+{X}_{i}$$

Assuming standard conditions (*P* = 1 atm, T = 293.15 K) and knowing the partial volume of CO_2_ in the samples (V_CO2_ = ppmv x 10^− 6^ * V_air_), the headspace volume (Sand: V_air_ = 107.5 mL; loam: V_air_ = 104.2 mL), and the universal gas constant (*R* = 0.0821 L atm K^− 1^ mol^− 1^) the ideal gas law was applied to convert ppmv into moles (n) per headspace. Using the molar mass of CO_2_ (M = 44.01 g mol^− 1^), the soil dry weight (m) and incubation time (t), the results were expressed according to Eq. 3 in µg CO_2_-C g^− 1^ soil h^− 1^ and thus comparable to the results obtained from the MicroResp method.

3 $$\:x=\frac{n*M}{t*m}$$

#### Scanning electron microscopy imaging

To qualitatively assess the effect of PAA on soil (micro)structural features scanning electron microscopy (SEM) images were taken with a FEI Quanta 250 ESEM (FEI Company Hillsboro, United States) using a secondary electron detector (SED) under high vacuum (< 10^− 4^ Pa). Prior to the measurements, the samples were coated with a 30 nm thick layer of gold with a Quorum Q150R S sputter coater (Quorom Technologies Ltd, United Kingdom). The air-dried soil samples of measurement points 0 and 10 (both cyclic and static), either untreated or at 2500 mg PAA kg^− 1^ dry soil, were exemplary selected for comparison. Different resolutions were used to visualize structural features at different scales, either for the overview of soil particle arrangement and PAA distribution or to detail interparticulate PAA hydrogel structures, particle bridges and altered surface morphology.

#### Statistical analysis

Data analysis was conducted using R version 4.3.0^31^, employing a comprehensive statistical approach to examine soil parameters and microbial responses. A three-way analysis of variance (ANOVA) was used to evaluate the effects of PAA concentration, experimental time, and soil moisture regimes (sample type) on WHC_max_, soil pH, and microbial respiration results (stats package). Statistical assumptions were tested including normality of residuals using Q-Q plots and Shapiro-Wilk tests (stats package), homogeneity of variances through Levene’s test (car package), and linearity via interaction plots. Post-hoc comparisons were performed using the Tukey HSD test (stats package). The F-value derived from the ANOVA represents the ratio of inter-group to intra-group variance, measuring the relative impact of each factor on the observed variables. It ranges from 0 to positive values, with higher F-values suggesting a stronger effect. The corresponding p-value assesses the significance of the respective effects, with *p* < 0.05 indicating statistical significance. Omega squared (ω²) was further calculated as an unbiased effect size estimate for each factor, ranging from 0 to 1, with higher values indicating a stronger influence of the independent variable on the dependent variable. For the headspace respiration data, a repeated measures ANOVA was applied (stats package), accounting for multiple measurements of the same sample over time.

A principal component analysis (PCA) was conducted to investigate relationships among soil physicochemical properties and microbial respiration variables (FactoMineR and factoextra packages). Continuous variables were standardized, and treatment conditions were incorporated as supplementary qualitative variables. The analysis was visualized through a biplot with 95% confidence interval ellipses, revealing complex interactions between experimental parameters.

Detailed statistical parameters, including F-values, p-values, and ω^2^, are presented in Tables S1-S3 of the supplementary information (SI).

## Results

### Maximum water holding capacity and soil pH

**Fig. 1:**
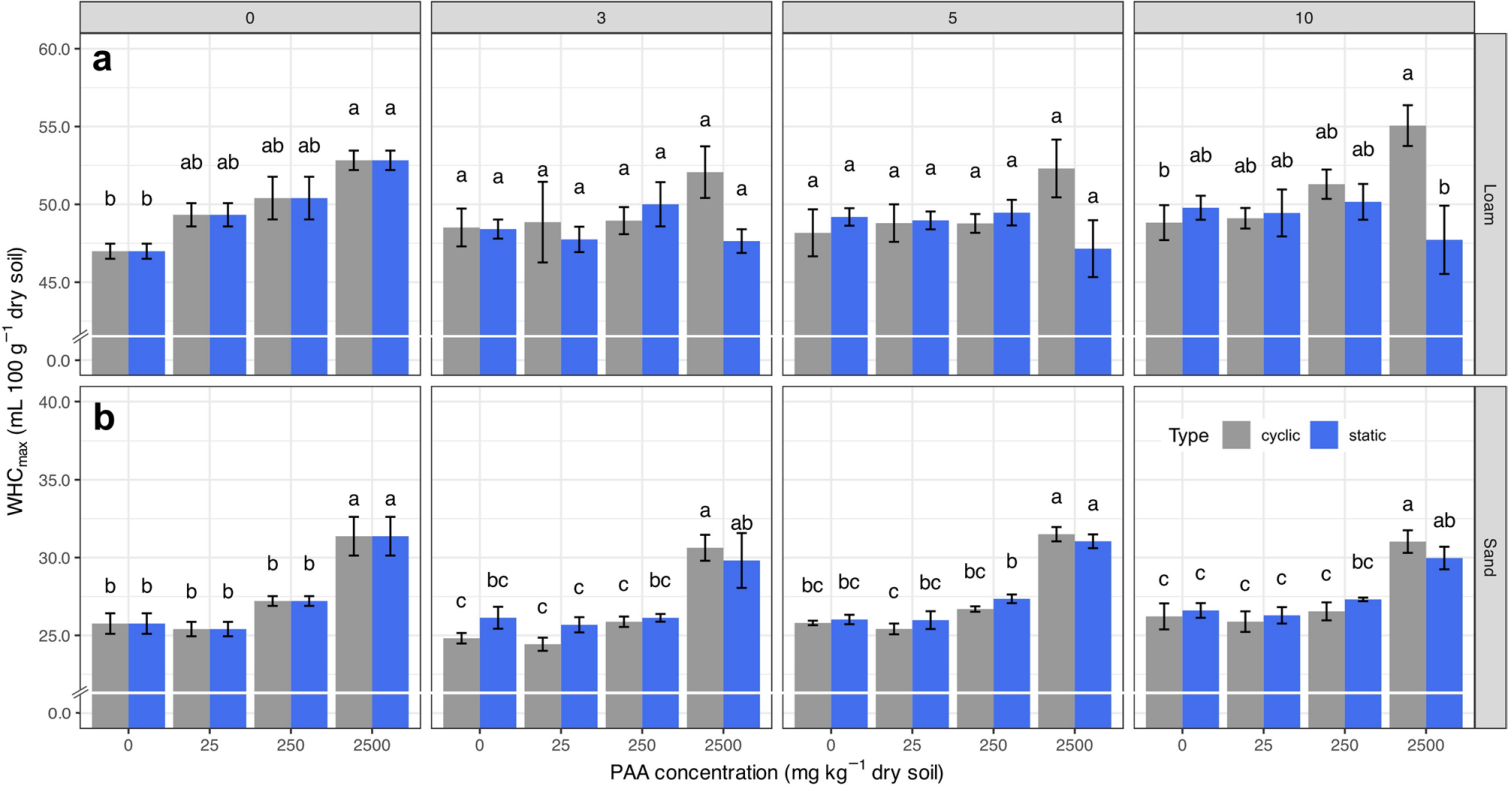
Maximum water holding capacity (WHC_max_) as function of PAA concentration (0–2500 mg kg^-1^ dry soil) and measurement point for (**a**) sand (Lufa 2.1) and (**b**) loam (Lufa 2.4). Numbers in the grey boxes denote the measurement point (incubation week/drying-rewetting cycle) for the static (blue bars) and cyclic soil samples (red bars), respectively. Different letters above the bars indicate significant differences between treatments (*p* < 0.05), based on the interaction between PAA concentration and moisture regime (conc × type). Error bars represent standard errors.

WHC_max_ measurements showed significant effects of PAA addition in both soils (Fig. 1 and Table 2a in the SI). The swelling capacity of freely swollen PAA in extracted soil solutions was 56 ± 0.2 mL g^− 1^ and 47 ± 0.2 mL g^− 1^ for the sand and loam, respectively, and was thus significantly lower than in demineralized water (76.8 ± 0.1 mL g^− 1^). For the sand, PAA concentration was the most significant factor (ω^2^ = 0.79) affecting the WHC_max_ variability (*p* < 0.00001, df = 3).

Already at measurement point 0, increasing PAA concentrations significantly increased WHC_max_ about 22%, from 25.8 ± 0.7 mL 100 g^− 1^ soil at 0 mg PAA kg^− 1^ dry soil to 31 ± 1 mL 100 g^−1^ soil at 2500 mg PAA kg^− 1^ dry soil. This effect persisted throughout the whole incubation time and for both cyclic and static soil moisture conditions. In addition, neither the incubation time, nor the number of drying-rewetting cycles, nor the soil moisture dynamics in general significantly affected the WHC_max_ of the sand.

For the loam, the PAA concentration (ω^2^ = 0.13; *p* = 0.0004, df = 3), the interaction between PAA concentration and sample type (ω^2^ = 0.12; *p* = 0.0006, df = 3), and sample type alone (ω^2^ = 0.03; *p* = 0.0032, df = 1) were the primary explanatory variables for WHC_max_ variability. At measurement point 0, PAA significantly increased WHC_max_ about 12.5%, from initially 47.0 ± 0.5 mL 100 g^− 1^ soil without PAA to 52.8 ± 0.6 mL 100 g^− 1^ soil at the highest PAA concentration. This increase remained significant under drying and rewetting, without further effects due to increasing cycles. Under static soil moisture conditions, the initial effect of PAA decreased over time, with no significant differences in WHC_max_ compared to the control by measurement point 3. Although not statistically significant, WHC_max_ showed a decreasing trend with increasing PAA concentrations in these weeks. Loam treated with the highest PAA concentration (2500 mg PAA kg^−1^ dry soil) and subjected to drying-rewetting cycles revealed a significantly higher WHC_max_ after 10 weeks compared to the respective soil incubated at constant soil moisture. When comparing the average increase in WHC_max_ between the control and the highest PAA concentration for the cyclic samples, no significant differences (*p* = 0.46) were observed between the sand (5.5 ± 0.2 mL 100 g^− 1^ soil) and the loam (4.9 ± 0.6 mL 100 g^− 1^ soil).

### Soil pH

**Fig. 2:**
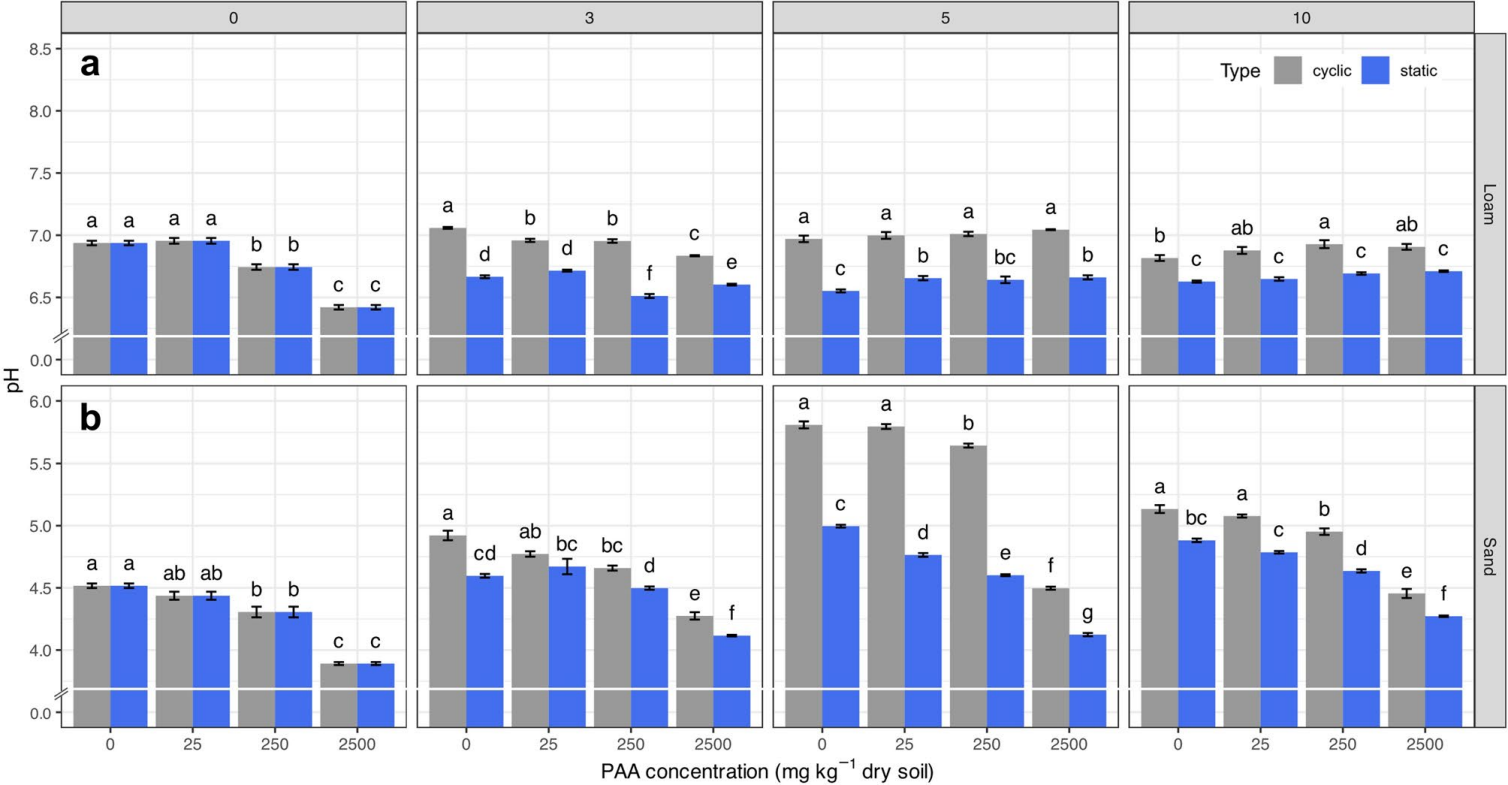
Soil pH as function of PAA concentration (0–2500 mg kg^− 1^ dry soil) and measurement point for (**a**) sand (Lufa 2.1) and (**b**) loam (Lufa 2.4). Numbers in grey boxes denote the measurement point (incubation week/drying-rewetting cycle) for the static (blue line) and cyclic soil samples (red line), respectively. Different letters above the bars indicate significant differences between treatments (*p* < 0.05), based on the interaction between PAA concentration and moisture regime (conc × type). Error bars represent standard errors.

PAA significantly (*p* < 0.001, df = 3) affected soil pH in both soils (Fig. 2 and Table 2b in SI). In the sand, PAA significantly reduced the soil pH directly after application from 4.52 ± 0.02 at 0 mg PAA kg^− 1^ dry soil to 3.89 ± 0.01 at 2500 mg PAA kg^− 1^ dry soil. Although the pH successively re-increased over time, its reduction remained significant. The sand subjected to drying-rewetting cycles showed significantly higher pH values at measurement point 3 and after compared to the samples incubated at constant moisture conditions. The most significant factors for soil pH variability were the PAA concentration (ω^2^ = 0.37) and the incubation time (ω^2^ = 0.34).

In the loam, PAA significantly reduced soil pH from initially 6.94 ± 0.02 at 0 mg PAA kg^− 1^ dry soil to 6.42 ± 0.02 at 2500 mg PAA kg^− 1^ dry soil. In contrast to the sand, the pH-reducing effect of PAA decreased over time, with pH values slightly (re)increasing from measurement point 5 until the end of the experiment. At measurement point 10, the soil pH at constant moisture conditions increased from initially 6.63 ± 0.01 at 0 mg PAA kg^−1^ dry soil to 6.71 ± 0.01 at 2500 mg PAA kg^− 1^ dry soil, whereas drying-rewetting cycles revealed pH changes from 6.82 ± 0.02 at 0 mg PAA kg^− 1^ dry soil to 6.91 ± 0.02 at 2500 mg PAA kg^− 1^ dry soil. Thus, the drying-rewetting cycles led to significantly higher pH values from measurement point 3 onwards than under static soil moisture conditions. The most significant factors for soil pH variability were soil moisture condition (ω^2^ = 0.40, *p* < 0.0001, df = 1) and the interaction of PAA concentration and time (ω^2^ = 0.30, *p* < 0.0001, df = 9). All in all, the sand exhibited a higher pH reduction in response to PAA addition compared to the loam. Furthermore, the pH reduction in the sand was significant throughout the entire incubation time, whereas the loam showed an acidifying effect over time. At the end of the experiment (measurement point 10), both soils subjected to drying-rewetting cycles showed significantly higher pH values compared to the respective soils incubated at static moisture conditions.

### Soil microbial activity and functional diversity

**Fig. 3:**
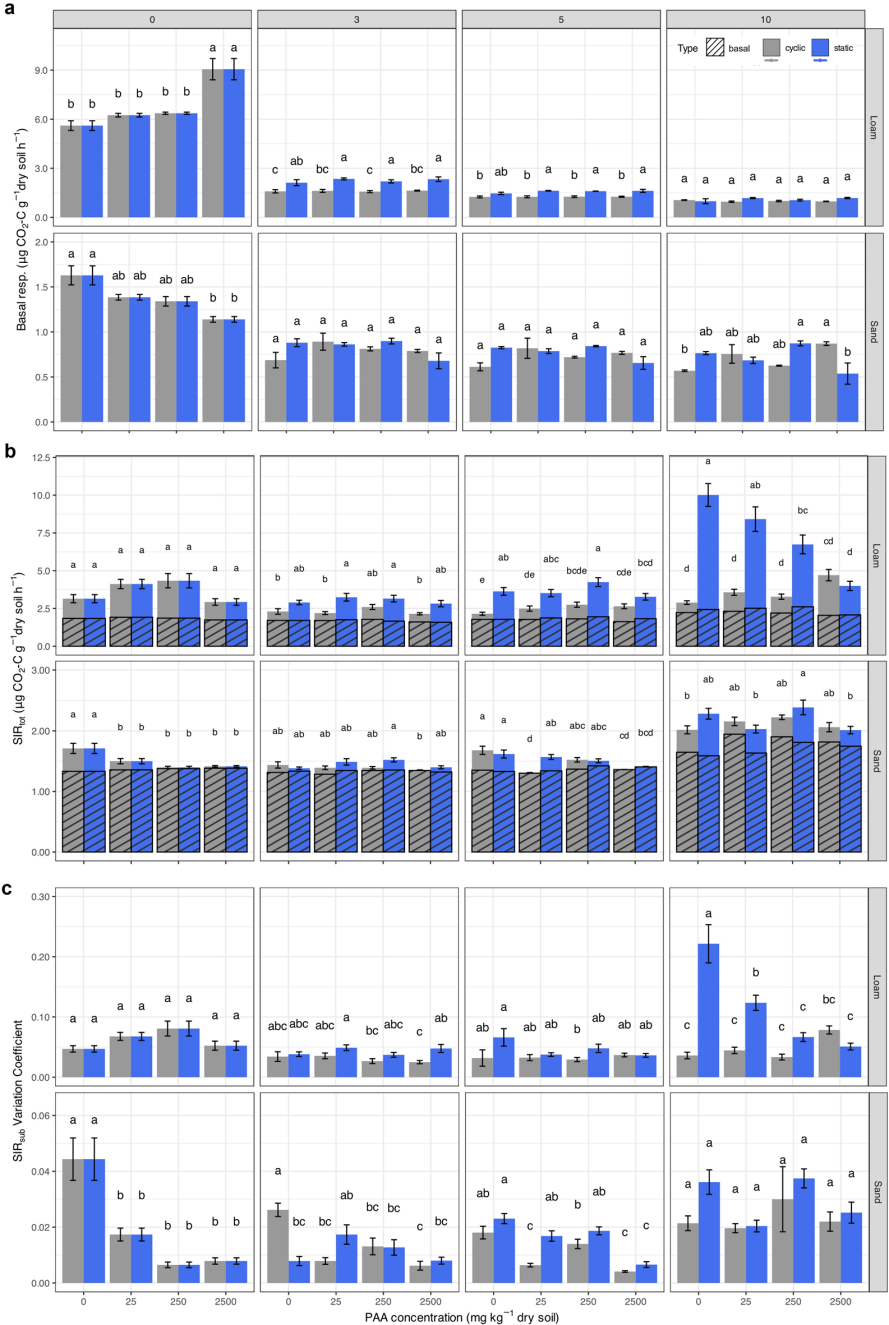
(**a**) Basal respiration for sand (Lufa 2.1) and loam (Lufa 2.4), (**b**) total substrate induced respiration (SIR_tot_), and (**c**) SIR_tot_ variation coefficient as a function of PAA concentration (0–2500 mg kg^− 1^ dry soil) and measurement point (incubation week/drying-rewetting cycle). Numbers in grey boxes denote the measurement point (incubation week/drying-rewetting cycle) for the static (blue line/bars) and cyclic soil samples (red line/bars), respectively. For SIR measurement, black-bordered bar areas represent basal respiration, grey-bordered areas indicate SIR fractions of SIR_tot_. Different letters above the bars indicate significant differences between treatments (*p* < 0.05), based on the interaction between PAA concentration and moisture regime (conc × type). Error bars represent standard errors.

Headspace CO_2_ measurements were performed to assess the basal respiration activity of the two investigated soils as function of the incubation time, soil moisture regimes and PAA concentration (Fig. 3a). On the one hand, regardless of the incubation conditions (constant soil moisture vs. drying-rewetting cycles) and PAA concentration, both soils showed the same trend in terms of a high CO_2_ release in the first week of incubation (measurement point 0), followed by a drastic drop and a constant respiration level from measurement point 3 onwards. Consequently, the incubation time was the primary factor (ω^2^ = 0.86, *p* < 0.0001, df = 3 for sand; ω^2^= 0.94, *p* < 0.0001, df = 3 for loam) explaining the variability in basal respiration of both soils (Table 3 in SI). In comparison, the sand showed lower basal respiration rates overall than the loam.

On the other hand, PAA reduced the basal respiration in the sand directly after addition (measurement point 0), from 1.6 ± 0.1 without PAA to 1.14 ± 0.03 µg CO_2_-C g^−1^ soil h^−1^ at the highest PAA concentration (−30 ± 2%). Interestingly, PAA increased the basal respiration in the loam, from 5.6 ± 0.3 without PAA to 9.1 ± 0.7 µg CO_2_-C g^−1^ soil h^−1^ at the highest PAA concentration (+ 62 ± 6%). However, the effect of PAA disappeared within the first three incubation weeks and was no longer significantly different after measurement point 3. Soil moisture regimes showed no significant effects on the basal respiration of both soils investigated, neither alone nor in combination with PAA.

In both soils, total substrate-induced respiration (SIR_tot_) significantly increased over time with the loam showing overall higher respiration rates than the sand (Fig. 3b; Table 4 in SI). Mean SIR_tot_ in the sand increased for all PAA concentrations and soil moisture regimes, from 1.51 ± 0.03 at measurement point 0 to 2.15 ± 0.04 µg CO_2_-C g^− 1^ soil h^− 1^ at measurement point 10. Mean SIR_tot_ in the loam increased from 3.6 ± 0.2 at measurement point 0 to 5.5 ± 0.4 µg CO_2_-C g^− 1^ soil h^− 1^ at measurement point 10. In both soils, the observed fluctuations in SIR_tot_ were predominantly attributed to changes in substrate-specific microbial responses rather than alterations in baseline microbial activity. This is evidenced by the relatively constant basal respiration across treatments, contrasting with the more pronounced and variable changes in SIR patterns (Fig. 3c).

**Fig. 4:**
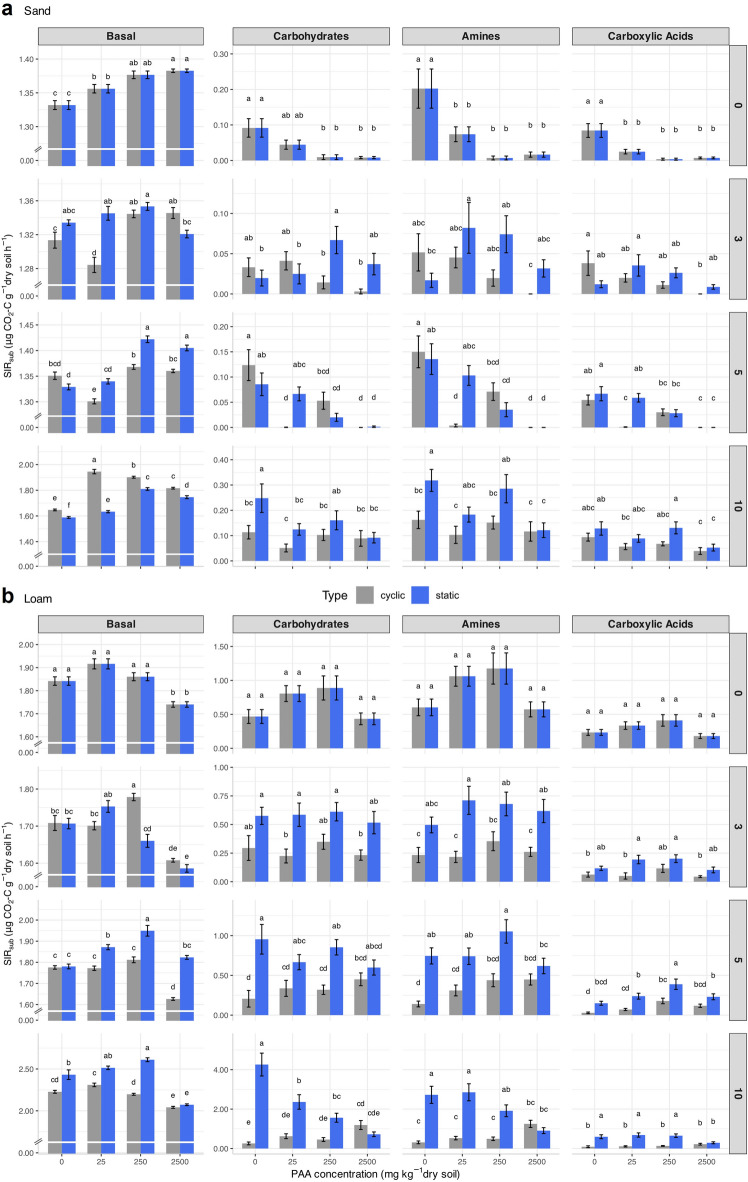
Basal and substrate-induced respiration (SIR) for (**a**) sand (Lufa 2.1) and (**b**) loam (Lufa 2.4) as a function of PAA concentration (0–2500 mg kg^−1^ dry soil) and measurement points (incubation week/drying-rewetting cycle) for the static (blue line) and cyclic soil samples (red line). Different letters above the bars indicate significant differences between treatments (*p* < 0.05), based on the interaction between PAA concentration and moisture regime (conc × type). Error bars represent standard errors.

For the sand, basal and substrate-induced respiration rates increased for all substrate groups (carbohydrates, amines, and carboxylic acids) over time, with incubation time being the most significant factor explaining the variability (ω² = 0.87, *p* < 0.0001, df = 3) (Fig. 4). PAA significantly increased basal respiration (ω^2^ = 0.03, *p* < 0.0001, df = 3), while it reduced SIR for carbohydrates (ω^2^ = 0.11, *p* < 0.0001, df = 3), amines (ω^2^ = 0.13, *p* < 0.0001, df = 3) and carboxylic acids (ω^2^ = 0.14, *p* < 0.0001, df = 3). These effects remained consistent throughout the incubation time. Compared to the untreated control soil, SIR reductions at the highest PAA concentration were 90%, 54%, 100%, and 52% at measurement points 0, 3, 5, and 10, respectively. Thus, the highest PAA concentration led to complete SIR suppression, particularly at measurement points 0 and 5. At the end of the incubation time, soil samples subjected to drying-rewetting cycles exhibited higher basal respiration rates (+ 7.28 ± 0.09%) than under static moisture conditions. Conversely, for all SIR groups, static soil moisture induced higher respiration rates than drying-rewetting cycles at PAA concentration up to 250 mg kg^−1^ dry soil (+ 85 ± 12%). At the highest PAA concentration, however, the two soil moisture regimes showed no significant differences anymore.

The SIR variation coefficients underscored these findings as they increased over time (ω^2^ = 0.12, *p* < 0.0001, df = 3) but decreased with higher PAA concentrations (ω^2^ = 0.14, *p* < 0.0001, df = 3), nearly dropping to zero during the initial weeks of incubation (Fig. 3c).

Comparable to the sand, both basal and substrate-induced respiration rates of the loam increased over time and for all substrate groups. However, in contrast to the sand, the basal respiration increased only at low PAA concentrations (ω^2^ = 0.08, *p* < 0.0001, df = 3), while the highest PAA concentration significantly reduced basal respiration. Comparing the control and the highest PAA concentration, the overall basal respiration decreased by 7.0 ± 0.3% at the beginning of the incubation for the SIR of carbohydrates, amines, and carboxylic acids. Interestingly, the effect of PAA addition on SIR was less consistent in the loam compared to the sand, with the two moisture regimes showing opposite responses: under drying-rewetting cycles, SIR respiration increased with increasing PAA concentration, whereas SIR respiration in static soil moisture decreased. Comparing the control and the highest PAA concentration, the overall SIR of the loam under static soil moisture conditions was reduced by 75 ± 1% after 10 weeks. Here, the highest respiration suppression of 83 ± 15% was observed for carbohydrates (4.3 ± 0.5 to 0.7 ± 0.1 µg CO_2_-C g^−1^ soil h^−1^). Although less pronounced, the same pattern was observed for amines and carboxylic acids.

From measurement point 5 on, the loam incubated at static moisture conditions consistently showed higher basal respiration rates compared to the respective samples subjected to drying-rewetting cycles. Also, the SIR rates were consistently higher from measurement point 3 onward at static soil moisture conditions than at drying-rewetting cycles. After 10 weeks, the loam incubated under static moisture conditions exhibited a 12.2 ± 0.1% increase in basal respiration and a 487 ± 62% increase in SIR when either untreated or treated with low PAA concentrations (up to 250 mg PAA kg^−1^ dry soil). As with the sand, the differences in loam decreased with increasing PAA concentration and for soil moisture regimes, with no significant differences anymore at the highest PAA concentration. The SIR variation coefficient for the loam further supports these findings, showing a significant increase over time (ω^2^ = 0.15, *p* < 0.0001, df = 3). While PAA concentration (ω^2^ = 0.02, *p* = 0.0005, df = 3) still slightly contributed to the variation, its effect was less pronounced than in the sand. In contrast, the sample type (ω^2^ = 0.06, *p* < 0.0001, df = 1) had a stronger influence on the variation in the loam, particularly towards the end of incubation, as demonstrated by its significant interaction with the incubation time (ω^2^ = 0.07, *p* < 0.0001, df = 3).

### Relationships between the parameters investigated

**Fig. 5:**
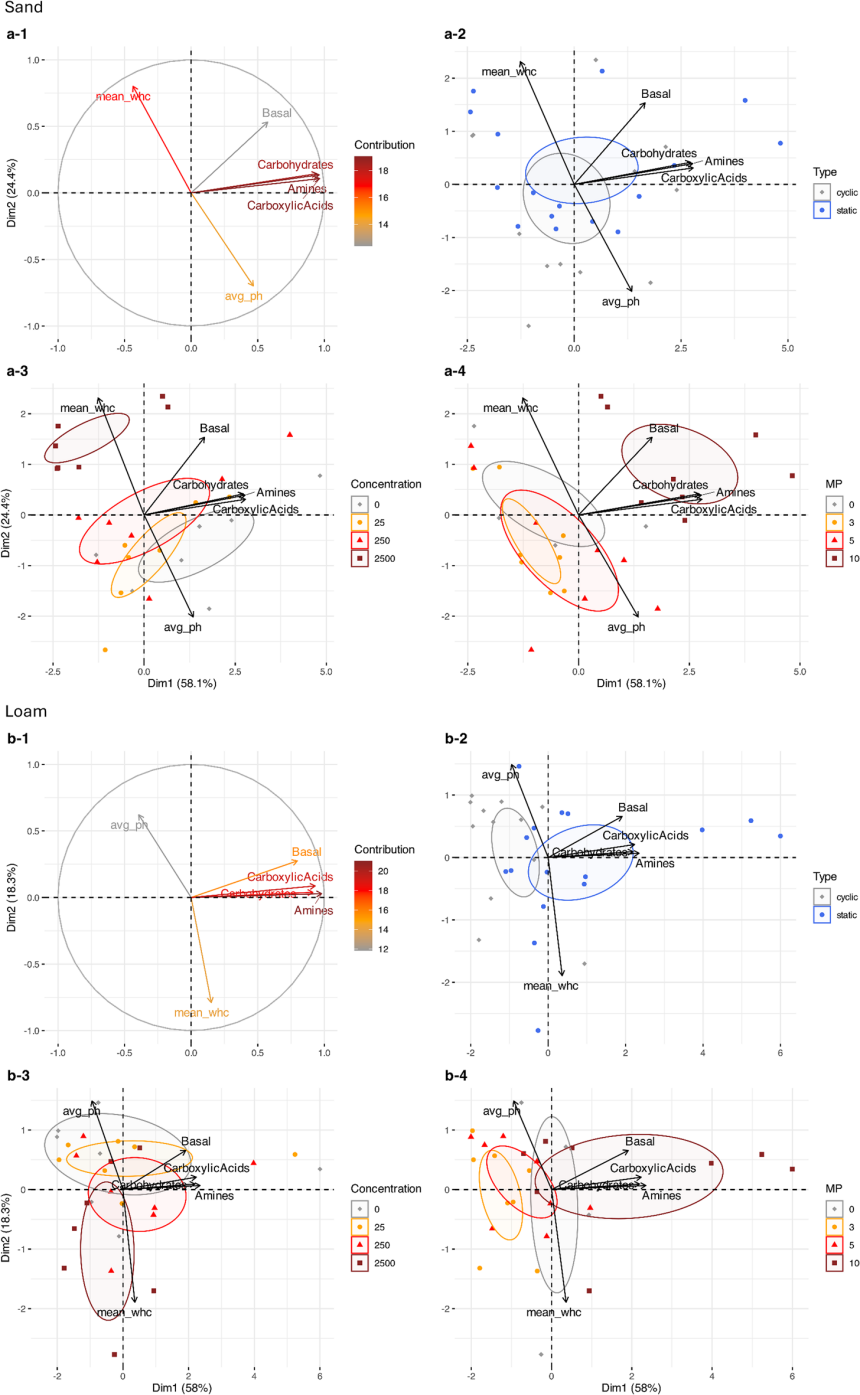
Principal component analysis (PCA) of the investigated parameters for (**a**) sand (Lufa 2.1) and (**b**) loam (Lufa 2.4) with (a-1; b-1) contribution of variables to PC1 and PC2, and biplots showing sample clustering by (a-2; b-2) soil moisture conditions (drying-rewetting cycles (**c**) and static moisture conditions (s)), (a-3; b-3) PAA concentration (0–2500 mg kg^-1^ dry soil), and (a-4; b-4)) measurement points (MP).

For the sand, PCA indicated that the first two principal components explained 82.5% of the total variance, with PC1 accounting for 58.1% and PC2 for 24.4% (Fig. 5a-1). PC1 was primarily influenced by SIR variables, including carboxylic acids, amines, and carbohydrates. PC2 was dominated by WHC_max_ and soil pH. A negative correlation was observed between soil pH and WHC_max_. SIR variables were positively correlated with each other and showed no correlation with WHC_max_ or soil pH.

The PCA biplot (Fig. 5a-2) revealed no distinct clustering based on the two soil moisture regimes: drying-rewetting cycles tended towards higher soil pH values, while static soil moisture conditions displayed a broader distribution along PC1 with higher respiration rates. Clustering based on PAA concentration (Fig. 5a-3) was more pronounced along PC2, where higher PAA concentrations were associated with increased WHC_max_ and lower soil pH values. Basal respiration rates did not show any clear clustering patterns between incubation conditions or substrate groups. However, for the SIR variables, the highest PAA concentration corresponded to the lowest respiration rates compared to the lower PAA concentrations and the control. The most distinct separation of samples was based on the measurement points, with measurement point 10 clearly separated from earlier points, indicating higher respiration rates along PC1 (Fig. 5a-4).

For the loam, Fig. 5b-1 indicated that the first two principal components explained 76.4% of the total variance, with PC1 accounting for 58.0% and PC2 for 18.3%. Similar to the sand, PC1 was dominated by SIR variables, while PC2 by WHC_max_ and soil pH. Again, soil pH was negatively correlated with WHC_max_, and the SIR variables were positively correlated with each other, showing no distinct relationship with WHC_max_ or soil pH. The PCA biplot (Fig. 5b-2) revealed distinct clustering based on the soil moisture regimes: soil samples subjected to drying-rewetting cycles tended to higher soil pH values, while static soil moisture conditions exhibited a broader distribution along PC1, with notably higher respiration rates. Clustering based on PAA concentration was apparent along PC2 (Fig. 5b-3), with higher PAA concentrations associated with increased WHC_max_ and reduced soil pH. At the highest PAA concentration, both basal respiration and SIR exhibited lower respiration rates and a narrower distribution along PC1 compared to the control and lower PAA concentrations. Clustering by measurement points (Fig. 5b-4) was distinct, with measurement point 10 clearly separated from earlier points, reflecting higher respiration rates along PC1. Over time, the cluster distribution shifted from a wider spread along PC2 to a broader distribution along PC1. This indicates that the influence of PAA concentration on soil pH and WHC_max_ of the loam diminished over time.

### Scanning electron microscopy

**Fig. 6:**
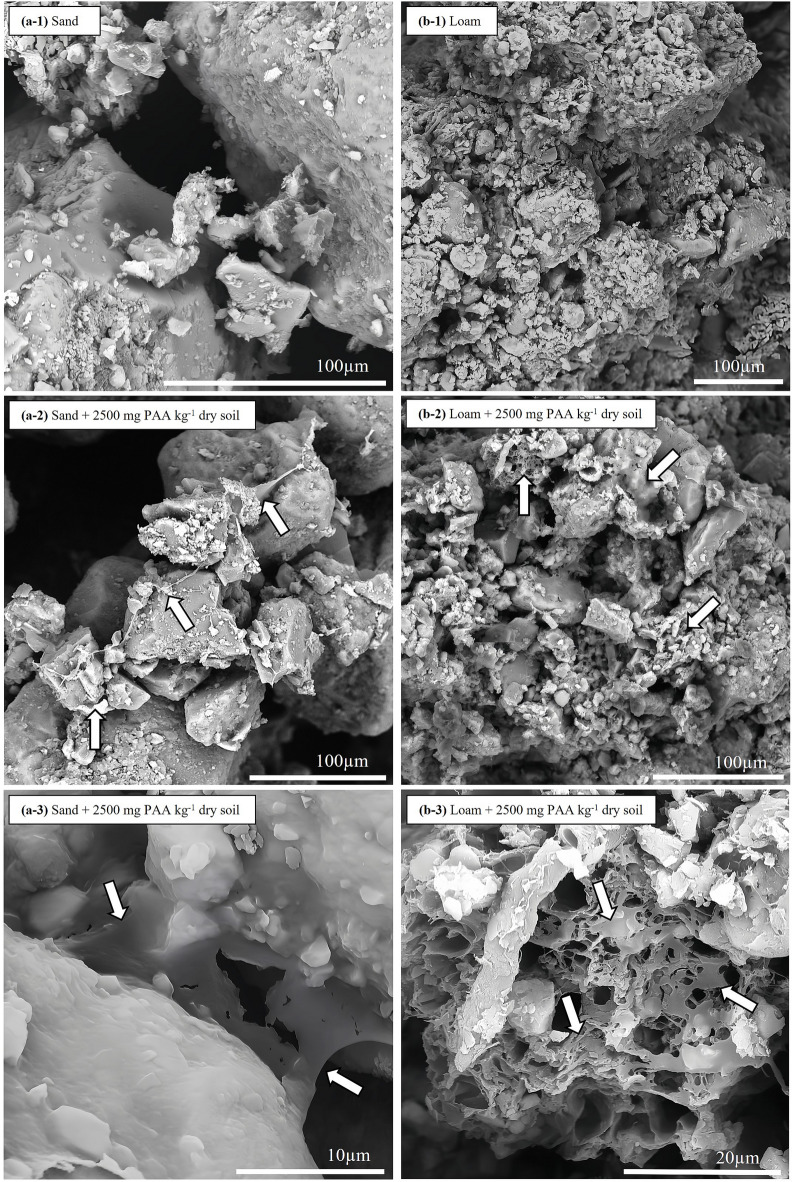
Scanning electron microscopy (SEM) images of (**a**) sand (Lufa 2.1) and (**b**) loam (Lufa 2.4), either (a-1; b-1) untreated or (a-2, a-3; b-2, b-3) PAA-treated (2500 mg kg^−1^ dry soil). Arrows highlight dehydrated PAA hydrogel structures bridging between soil particles or OM structures.

Although the SEM images did not show noticeable differences based on measurement point or soil moisture regimes for both soils, they clearly showed membranous PAA structures between the soil particles, forming coatings as well as interparticulate bridges and connections of varying sizes (Fig. 6). These structures were visible in both sand and clay and covered not only individual particles, smaller particle clusters and aggregate surfaces, but also extended over larger interparticle spaces, especially at the highest PAA concentration. At higher magnification, these structures became even clearer, showing the PAA membrane network connected not only soil particles but also organic material with both smooth, compact membrane zones as well as areas with filament- and honeycomb-like features.

## Discussion

The interplay between PAA application and soil moisture regimes revealed reciprocal, soil-specific effects on WHC_max_ and soil pH that subsequently influenced microhabitat conditions and altered soil microbial activity, particularly substrate use efficiency and respiration dynamics.

As hypothesized, PAA significantly increased WHC_max_ by up to 22% in the sand and 12.5% in the loam at the highest application rate and directly at the beginning of the incubation period. This is in line with various other studies investigating the effect of SAPs on soil WHC_max_, although the effect size partly differed considerably^32–34^. For example, Bhardwaj et al.^34^ investigated four crosslinked acrylamide and acrylic acid polymers in a sandy soil with similar grain size distribution, CEC, and OM content, but different application types (powder vs. granules of up to 2 mm). Here, PAA applied at a concentration of 2500 mg kg^− 1^ dry soil increased the water content of the sandy soil by roughly 65%, corresponding to an absorption capacity of roughly 90 mL water g^− 1^ polymer. In our study, the PAA powder only absorbed 22 mL water g^− 1^ in the sand, highlighting the effect of the application form on its efficiency to improve soil water retention: PAA granules with their the relatively lower surface area compared to PAA powder facilitate the formation of a more stable, localized hydrogel network that can effectively counteract the confining pressure and suction tension of the soil matrix^35,36^. In contrast, PAA powder provides a relatively high surface area, which promotes strong and fast interactions with soil particles, e.g., adsorption processes, coming along with its reduced swelling potential^5^. Furthermore, the homogeneous mixing of PAA powder into the soils likely restricted the formation of locally distinct, highly cohesive hydrogel patches that resisted the confining pressure of the soil matrix to efficiently absorb water from its surrounding^27,37^.

For both investigated soils, the PAA-induced effects on WHC_max_ showed a time-, concentration-, and moisture regime-dependent behavior. However, the results on free PAA swelling further revealed a soil type-dependent effect strength in soil, as the swelling capacity of PAA was lower in both soil solutions compared to demineralized water. This is in line with the results of Buchmann et al.^5,27^, who showed that the soil solution composition significantly determines the swelling potential and the resulting physicochemical properties of PAA both freely swollen and in the soil interparticle space. Furthermore, Bai et al.^38^ showed that SAP swelling further decreased in ultrapure water, tap water, and soil extracts with increasing drying-rewetting cycles, with the degree of functional loss directly related to the severity of the drying events. In the investigated sand, the observed functional resilience of PAA can be attributed to the relatively loose soil texture and the high proportion of coarse soil pores that enable an easier and less restricted interparticulate hydrogel (re)expansion after drying. In addition, its relatively low pH and CEC, as well as the rather weak and few polymer-mineral interactions allowed a relatively undisturbed PAA (re)swelling in the sand with minimal structural resistance^39,40^.

Contrary to the sand, PAA in the loam completely lost its functionality over time, with WHC_max_ dropping even below control levels. Notably, this decline was most pronounced at measurement points 5 and 10 under static moisture conditions and the highest PAA concentration. In this regard, freely swollen PAA also exhibited the highest swelling reduction in soil solution of loam compared to sand or demineralized water. These effects likely resulted from cation-mediated crosslinking between PAA polymer chains, solution salinity, and the low soil pH, all of them typically restricting hydrogel swelling^7,8,16^. On the one hand, the high ionic strength in the soil solution reduces the osmotic gradient between PAA and the solution and, together with the water (re)distribution over time or during the drying-wetting cycles, leads to a successive, gradual dehydration of PAA in the soil interparticle space until reaching equilibrium conditions with the surrounding^27,37,41–43^. On the other hand, soil pH in both soils decreased immediately after PAA addition, due to the dissociation of carboxylic acid groups, which release hydrogen ions and mobilize cations from exchange sites^44^. This process facilitated crosslinking both within the PAA hydrogel network and with mineral surfaces, thereby progressively limiting further PAA swelling^45,46^. PCA results of both soils supported these observations, with WHC_max_ strongly aligned along PC2 and inversely related to pH, underlining the mechanism by which PAA swelling induces hydrogen ion release through carboxyl group dissociation, leading to acidification of the surrounding solution and simultaneously promoting cross-linking that gradually limits further PAA swelling. However, this effect persisted only in the sand and disappeared in the loam already after one week. Here, the favorable pH buffer conditions in terms of higher CEC and C_org_ in the loam (three times that of sand) likely restabilized the soil pH, e.g., through SOM-related functional groups or ion exchange at clay mineral surfaces over time^47–49^.

The results also suggest that drying–wetting cycles partly counteracted restricted PAA (re)swelling by intermittently disrupting prolonged polymer–clay contact, thereby slowing irreversible dehydration and preserving rewettable interfaces^6^. This interpretation is further supported by Buchmann et al.^5^ and Vu et al.^50^, who showed a remaining or prolonged degree of (re)swelling, although transient drying–rewetting events reduced polymer–clay interactions and interrupted water (re)distribution in the investigated, fine-textured soils. Further, Hafidi et al.^51^ showed that hydrogel crosslinking via cations such as Cu^2+^ and Zn^2+^ can occur within hours, indicating that PAA (re)swelling during drying–rewetting cycles is likely governed less by inhibited crosslinking and more by the temporary loss of existing polymer–soil interactions.

The results and PCA analyses, with PC1 aligned with microbial respiration and PC2 with WHC_max_ and pH, highlight a reciprocal interaction between soil texture, soil solution, and PAA swelling, indicating that PAA-induced shifts in WHC_max_ and pH alter soil aggregation, pore structure, and water dynamics, ultimately reshaping microhabitats and microbial activity^15,52,53^. As already indicated, drying–rewetting cycles modulated polymer–clay interactions, with the higher CEC and pH buffering of the loam promoting rapid PAA crosslinking via polyvalent cations, forming organo-mineral complexes that limited PAA (re)swelling in the soil interparticle space. Consequently, the cohesive PAA linkages between the soil particles markedly reduced effective soil porosity, particularly at higher PAA concentrations and during drying, when interconnected hydrogel domains experienced cumulative suction stress. In this regard, various studies already demonstrated that drying events in PAA-treated soils resulted in dense organo-mineral complexes in terms of cemented and membranous polymer structures that occupied large parts of the soil interparticle space^5,37,54^. In their experiments, the total pore volume remained constant, but the pore systems shifted towards smaller, strongly water-retaining soil pores, reducing water availability and (air-filled).

For the untreated control soils, the measured basal respiration were within the range reported in other studies, with a tendency for higher respiration rates observed under static moisture conditions compared to drying-rewetting conditions^55–58^. Repeated drying-rewetting cycles typically impose osmotic and oxidative stress on soil microbial communities, leading to cell lysis and reducing their biomass with significant shifts in microbial energy use. In this case, soil microorganisms divert resources toward stress recovery mechanisms, including cell repairing or osmolyte production instead of respiration^59–61^. This stress also tends to reduce microbial diversity, with stress-tolerant microorganisms becoming more dominant. Conversely, under static moisture conditions, soil microbial communities experience fewer disruptions, allowing them to maintain a more stable environment^62,63^. This stability enables higher metabolic activity and basal respiration rates, as they can focus more energy on growth and maintenance rather than stress recovery.

Concerning the differences in basal respiration as function of soil type, the loam consistently showed higher microbial respiration rates than the sand, which aligns with existing literature linking basic soil physicochemical properties such as soil pH, SOM content and soil pore water availability to soil respiration^64,65^. One key factor explaining the differences is the pH, a well-established determinant of microbial activity and community composition. Jones et al.^21^ identified a critical threshold at pH 5.5, below which microbial carbon use efficiency and respiration tend to decline. While the loam consistently maintained pH values above this threshold, regardless of the PAA concentration, the sand exhibited overall lower pH values, likely contributing to its reduced soil microbial activity and respiration. This could also explain the relatively lower SIR response to added carbon sources in the sand compared to the loam, respectively. Also in line with Jones et al.^21^, the results suggest that the soil microbial community in the loam exhibited a higher capacity to decompose the added substrates, whereas it was less responsive to changes in resource availability in the sand. Yazdanpanah^66^ found similar results, observing a stronger increase in respiration with rising carbon content in a clay loam compared to a loamy sand.

Contrary to our expectations and current scientific knowledge, PAA neither showed a clear drought-mitigating effect in the two investigated soils, nor did it lose its effects on soil microbial respiration over time or due to moisture dynamics. In the loam, SIR was reduced by up to 83% for carbohydrates under static moisture conditions and at 2500 mg PAA kg⁻¹ dry soil, particularly at later stages of incubation. Also in the sand, SIR values for carbohydrates and amines significantly dropped at the highest PAA concentration under both moisture regimes by measurement point 5, indicating severe microbial suppression and a decreased capacity to utilize added carbon sources. Since PAA is well-known to be mostly non-biodegradable^9^, the effects on soil microbial activity did not result from to the utilization of PAA-derived carbon. In nature and as shown by the SIR results, the reduced utilization of easily degradable carbohydrates, especially at high PAA concentrations, suggests enzyme inhibition, such as of amylases, e.g., due to a decreased abundance or activity of carbohydrate-utilizing microorganisms. For more complex substrates like amines and carboxylic acids, which require specialized metabolic pathways (e.g., deamination for amines and beta-oxidation for carboxylic acids), the reduced utilization suggests similar inhibitory effects at high PAA concentrations. Potential mechanisms include oxidative stress and PAA-induced shifts in soil that suppress soil microbial metabolism, as already shown for other synthetic polymers such as polyethylene and polylactic acid^67,68^. This, in turn, can shift soil microbial community structures towards microorganisms favoring simpler substrates over those capable of degrading more complex compounds. Also, changes in soil pH due to PAA deprotonation directly influence soil microbial activity, as certain microbial groups are more adapted to wide pH ranges or resilient to rapid pH changes^69–71^. Thus, pH shifts might favor or inhibit acidophilic or alkaliphilic microorganisms, potentially disrupting the overall balance of the soil microbial community and impairing their ability to process OM^72–74^.

Concerning the additional effect of soil moisture conditions, the results showed their substantial contribution to soil microbial respiration, as both excessive and insufficient water availability can reduce soil microbial activity^75,76^. High PAA concentrations or soil moisture can limit both oxygen and nutrient availability by restricting aerobic respiration^77^, a process that can be further intensified when swollen PAA hydrogel partly or even completely occupies the soil interparticle space and thereby reduces soil permeability and soil pore interconnectivity^78,79^. This, in turn, restricts the (re)distribution of soil pore water and dissolved substrates, limiting the ability of soil microorganisms to access nutrients and labile carbon^80^. Over time, especially under drying–rewetting cycles, the formation of condensed, semi-permeable PAA residues likely intensified these effects by (irreversibly) cementing soil particles and partly clogging the soil interparticle space, reducing resource availability and resulting in more stress-tolerant, slow-growing soil microorganisms with inherently lower respiration rates and increased fungi-to-bacteria ratios^81,82^. PAA-induced soil structural changes were further supported by previous studies^5,50,83^ and by the conducted ESEM measurements, which revealed solid, membrane-like PAA structures in soil that coated both mineral and OM surfaces. In this regard, the potential of PAA to form plastic-like, persistent residues in soil that can irreversibly alter soil physicochemical properties and soil functions is receiving increasing attention, which is particularly underlined by recently reviewed literature^3,6^ and the results shown here.

Although our study provided valuable first insights into the effect of PAA on soil microbial activity and substrate utilization, there are several issues that should be further explored: one point is the limited diversity of substrates used in MicroResp studies, which may not fully reflect the range of OM utilized by soil microorganisms. Thus, including a broader variety of substrates could provide a more comprehensive view of microbial processes under PAA exposure. Additionally, while shifts in soil microbial community composition were suggested, detailed profiling of soil microbial groups and their functional pathways is still lacking, which could be addressed using advanced molecular techniques, such as metagenomics^84^. Although two soils have been investigated in this study, the variability in soil types and long-term effects of PAA exposure also remain underexplored, highlighting the need to conduct systematic studies with different soil types and implementing long-term exposure assessments. Furthermore, environmental and agricultural factors (e.g., climate and soil management) determining the fate of PAA and its effects on soil physicochemical properties need more attention in targeted multi-factor studies^6^. Finally, the exact mechanisms behind the ecotoxicological impact of PAA on soil microbiology, e.g., enzyme inhibition or oxidative stress, require further targeted biochemical investigations^85^. Thus, future work should particularly focus on the spatial- and time-resolved interactions between structural dynamics (e.g., soil pore size shifts, soil aggregation dynamics), PAA transformation behavior, and functional soil microbial responses (e.g., EPS production and stress gene expression).

## Conclusion

This study demonstrated that PAA application significantly alters soil physicochemical properties in terms of WHC_max_ and soil pH, and soil microbial activity as function of soil texture, application rate, and moisture regime. PAA persistently increased WHC_max_ in sand, while in loam, its effect diminished under static moisture conditions but was partially preserved under drying–rewetting cycles. PAA-induced soil acidification was soil-specific and transient, attenuated by the buffering capacity of the loam and the imposed moisture dynamics. In addition, PAA induced distinct soil structural changes by forming membranous hydrogel structures between soil particles, which modulated effective soil porosity, especially under static moisture conditions. These structural modifications constrained oxygen diffusion, water (re)distribution, and substrate accessibility, thereby directly modulating the soil microhabitat and microbial respiration.

All in all, PAA-induced effects on soil microbial activity exhibited texture- and moisture-specific patterns: in sand, high PAA concentrations strongly suppressed substrate-induced respiration regardless of moisture regime, suggesting long-term reductions in soil microbial functional diversity. In loam, soil microbial responses diverged under different moisture treatments, with drying–rewetting cycles partially mitigating the suppressive effects of PAA on soil microbial functioning.

Although this study highlights the significant role of environmental and physicochemical stressors in shaping the effects of PAA application on soil microbial activity, it also underscores the need for a broader evaluation of long-term soil impacts. As the soil type modulated the magnitude and persistence of PAA-induced effects, and considering the widespread SAP utilization in agriculture and land management, a more comprehensive understanding of its cumulative and long-term effects on soil microbial communities, nutrient cycling, and ecosystem stability is essential. Future research should therefore investigate soil microbial adaptation mechanisms, the (long-term) persistence and transformation of PAA in soil, and potential mitigation strategies to balance the benefits of SAPs with their potential risks to soil health and ecosystem functioning.

## Electronic supplementary material

Below is the link to the electronic supplementary material.

Supplementary Material 1

Supplementary Material 2

Supplementary Material 3

## Data Availability

The data that supports the findings of this study are available from the corresponding author upon reasonable request.
