# Supplementary material for: Impact of polyacrylic acid as soil amendment on soil microbial activity under different moisture regimes
Source: Sci Rep. 2025 Jun 3;15:19422. doi: 10.1038/s41598-025-04457-8 (PMC12134354; doi:10.1038/s41598-025-04457-8)
Supplement: Supplementary file 1 — Supplementary Material 1 [file 41598_2025_4457_MOESM1_ESM.docx]

**Table S1** Analysis of variance (ANOVA) of a) WHC_max_ and b) soil pH for sand (Lufa 2.1) and loam (Lufa 2.4) as function of PAA concentration (conc), incubation time (week), moisture conditions (type). Significant effects and interactions are shown marked in bold, respectively

| **a** WHC_max_ | | | | | | | | | | | | | | | | | | | | | | | | | | | | |
| --- | --- | --- | --- | --- | --- | --- | --- | --- | --- | --- | --- | --- | --- | --- | --- | --- | --- | --- | --- | --- | --- | --- | --- | --- | --- | --- | --- | --- |
|  |  | Lufa 2.1 | | | | | | | | | | | | | | | Lufa 2.4 | | | | | | | | | | | |
|  | | | | Df | | Sum Sq | | Mean Sq | | F value | | Pr(>F) | | ω^2^ | | Df | | | Sum Sq | | Mean Sq | | F value | | Pr(>F) | | ω^2^ | |
| conc | | | | 3 | | 427.50 | | 142.50 | | 110.99 | | **0.0000** | | **0.79** | | 3 | | | 93.35 | | 31.12 | | 7.07 | | **0.0004** | | **0.13** | |
| week | | | | 3 | | 10.87 | | 3.62 | | 2.82 | | **0.0457** | | **0.01** | | 3 | | | 23.52 | | 7.84 | | 1.78 | | 0.1598 | | 0.02 | |
| type | | | | 1 | | 1.14 | | 1.14 | | 0.89 | | 0.3496 | | 0.00 | | 1 | | | 21.16 | | 21.16 | | 4.81 | | **0.0320** | | **0.03** | |
| conc:week | | | | 9 | | 6.37 | | 0.71 | | 0.55 | | 0.8310 | | 0.01 | | 9 | | | 46.61 | | 5.18 | | 1.18 | | 0.3253 | | 0.01 | |
| conc:type | | | | 3 | | 5.18 | | 1.73 | | 1.35 | | 0.2676 | | 0.00 | | 3 | | | 87.75 | | 29.25 | | 6.64 | | **0.0006** | | **0.12** | |
| week:type | | | | 3 | | 0.82 | | 0.27 | | 0.21 | | 0.8871 | | 0.01 | | 3 | | | 10.01 | | 3.34 | | 0.76 | | 0.5219 | | 0.01 | |
| conc:week:type | | | | 9 | | 3.44 | | 0.38 | | 0.30 | | 0.9729 | | 0.02 | | 9 | | | 40.37 | | 4.49 | | 1.02 | | 0.4348 | | 0.00 | |
| Residuals | | | | 64 | | 82.17 | | 1.28 | |  | |  | |  | | 64 | | | 281.76 | | 4.40 | |  | |  | |  | |
| **b** Soil pH | | | | | | | | | | | | | | | | | | | | | | | | | | | |  |
| conc | | | 3 | | 7.78 | | 2.59 | | 1380.97 | | **0.0000** | | **0.37** | | 3 | | | 0.29 | | 0.10 | | 96.52 | | **0.0000** | | **0.09** | |  |
| week | | | 3 | | 7.14 | | 2.38 | | 1266.15 | | **0.0000** | | **0.34** | | 3 | | | 0.04 | | 0.01 | | 12.03 | | **0.0000** | | **0.01** | |  |
| type | | | 1 | | 2.39 | | 2.39 | | 1272.81 | | **0.0000** | | **0.11** | | 1 | | | 1.27 | | 1.27 | | 1258.22 | | **0.0000** | | **0.40** | |  |
| conc:week | | | 9 | | 0.67 | | 0.07 | | 39.45 | | **0.0000** | | **0.03** | | 9 | | | 0.97 | | 0.11 | | 107.22 | | **0.0000** | | **0.30** | |  |
| conc:type | | | 3 | | 0.15 | | 0.05 | | 27.16 | | **0.0000** | | **0.01** | | 3 | | | 0.02 | | 0.01 | | 5.60 | | **0.0018** | | **0.00** | |  |
| week:type | | | 3 | | 2.22 | | 0.74 | | 393.23 | | **0.0000** | | **0.11** | | 3 | | | 0.51 | | 0.17 | | 168.56 | | **0.0000** | | **0.16** | |  |
| conc:week:type | | | 9 | | 0.34 | | 0.04 | | 20.40 | | **0.0000** | | **0.02** | | 9 | | | 0.04 | | 0.00 | | 4.45 | | **0.0001** | | **0.01** | |  |
| Residuals | | | 64 | | 0.12 | | 0.00 | |  | |  | |  | | 64 | | | 0.06 | | 0.00 | |  | |  | |  | |  |
