# Supplementary material for: Impact of polyacrylic acid as soil amendment on soil microbial activity under different moisture regimes
Source: Sci Rep. 2025 Jun 3;15:19422. doi: 10.1038/s41598-025-04457-8 (PMC12134354; doi:10.1038/s41598-025-04457-8)
Supplement: Supplementary file 2 — Supplementary Material 2 [file 41598_2025_4457_MOESM2_ESM.docx]

**Table S2** Analysis of variance (Repeated Measures ANOVA) results of the basal soil respiration for sand (Lufa 2.1) and loam (Lufa 2.4) as function of PAA concentration (conc), incubation time (week), moisture conditions (type). Significant effects and interactions are marked in bold, respectively

|  |  | Lufa 2.1 | | | | | | | | Lufa 2.4 | | | | | |
| --- | --- | --- | --- | --- | --- | --- | --- | --- | --- | --- | --- | --- | --- | --- | --- |
| Error: ID | | | | | | | | | | | | | | | |
|  | | | Df | Sum Sq | Mean Sq | F value | Pr(>F) | ω^2^ | Df | | Sum Sq | Mean Sq | F value | Pr(>F) | ω^2^ |
| conc | | | 3 | 0.27 | 0.09 | 5.116 | **0.0114** | **0.23** | 3 | | 11.80 | 3.93 | 28.23 | **0.0000** | **0.71** |
| type | | | 1 | 0.01 | 0.01 | 0.742 | 0.4017 | 0.00 | 1 | | 1.71 | 1.71 | 12.25 | **0.0030** | **0.10** |
| conc:type | | | 3 | 0.32 | 0.11 | 6.241 | **0.0052** | **0.29** | 3 | | 0.10 | 0.03 | 0.24 | 0.8702 | 0.00 |
| Residuals | | | 16 | 0.28 | 0.02 |  |  |  | 16 | | 2.30 | 0.14 |  |  |  |
| Error: ID:week | | | | | | | | | | | | | | | |
|  | | | Df | Sum Sq | Mean Sq | F value | Pr(>F) | ω^2^ | Df | | Sum Sq | Mean Sq | F value | Pr(>F) | ω^2^ |
| week | | | 3 | 6.93 | 2.31 | 297.54 | **0.0000** | **0.86** | 3 | | 525.4 | 175.12 | 1685.35 | **0.0000** | **0.94** |
| conc:week | | | 9 | 0.60 | 0.07 | 8.57 | **0.0000** | **0.07** | 9 | | 30.4 | 3.38 | 32.52 | **0.0000** | **0.05** |
| type:week | | | 3 | 0.01 | 0.00 | 0.38 | 0.7710 | 0.00 | 3 | | 1.5 | 0.49 | 4.70 | **0.0059** | **0.00** |
| conc:type:week | | | 9 | 0.18 | 0.02 | 2.52 | **0.0188** | **0.01** | 9 | | 0.0 | 0.01 | 0.05 | 0.9999 | 0.00 |
| Residuals | | | 48 | 0.37 | 0.01 |  |  |  | 48 | | 5.0 | 0.10 |  |  |  |
