# Supplementary material for: Impact of polyacrylic acid as soil amendment on soil microbial activity under different moisture regimes
Source: Sci Rep. 2025 Jun 3;15:19422. doi: 10.1038/s41598-025-04457-8 (PMC12134354; doi:10.1038/s41598-025-04457-8)
Supplement: Supplementary file 3 — Supplementary Material 3 [file 41598_2025_4457_MOESM3_ESM.docx]

**Table S3** Analysis of variance (ANOVA) results of basal and substrate induced respiration (SIR) for sand (Lufa 2.1) and loam (Lufa 2.4) as function of PAA concentration (conc), incubation time (week), moisture conditions (type), and subdivided for the different substance groups investigated (carbohydrates, amines, carboxylic acid). Significant effects and interactions are marked in bold, respectively

|  |  | Lufa 2.1 | | | | | | | | Lufa 2.4 | | | | | |
| --- | --- | --- | --- | --- | --- | --- | --- | --- | --- | --- | --- | --- | --- | --- | --- |
| Basal | | | | | | | | | | | | | | | |
|  | | | Df | Sum Sq | Mean Sq | F value | Pr(>F) | ω^2^ | Df | | Sum Sq | Mean Sq | F value | Pr(>F) | ω^2^ |
| conc | | | 3 | 0.43 | 0.14 | 234.28 | **0.0000** | **0.03** | 3 | | 2.27 | 0.76 | 185.96 | **0.0000** | **0.08** |
| week | | | 3 | 12.20 | 4.07 | 6608.50 | **0.0000** | **0.87** | 3 | | 21.01 | 7.00 | 1723.35 | **0.0000** | **0.77** |
| type | | | 1 | 0.05 | 0.05 | 74.76 | **0.0000** | **0.00** | 1 | | 0.54 | 0.54 | 133.01 | **0.0000** | **0.02** |
| conc:week | | | 9 | 0.45 | 0.05 | 80.84 | **0.0000** | **0.03** | 9 | | 0.70 | 0.08 | 19.16 | **0.0000** | **0.02** |
| conc:type | | | 3 | 0.03 | 0.01 | 17.11 | **0.0000** | **0.00** | 3 | | 0.06 | 0.02 | 4.66 | **0.0033** | **0.00** |
| week:type | | | 3 | 0.41 | 0.14 | 219.47 | **0.0000** | **0.03** | 3 | | 0.85 | 0.28 | 69.58 | **0.0000** | **0.03** |
| conc:week:type | | | 9 | 0.27 | 0.03 | 49.00 | **0.0000** | **0.02** | 9 | | 0.59 | 0.07 | 16.12 | **0.0000** | **0.02** |
| Residuals | | | 352 | 0.22 | 0.00 |  |  |  | 352 | | 1.43 | 0.00 |  |  |  |
| Carbohydrates | | | | | | | | | | | | | | | |
|  | | | Df | Sum Sq | Mean Sq | F value | Pr(>F) | ω^2^ | Df | | Sum Sq | Mean Sq | F value | Pr(>F) | ω^2^ |
| conc | | | 3 | 0.26 | 0.09 | 25.63 | **0.0000** | **0.11** | 3 | | 6.53 | 2.18 | 10.70 | **0.0000** | **0.02** |
| week | | | 3 | 0.53 | 0.18 | 52.39 | **0.0000** | **0.22** | 3 | | 59.35 | 19.78 | 97.22 | **0.0000** | **0.20** |
| type | | | 1 | 0.04 | 0.04 | 11.51 | **0.0008** | **0.02** | 1 | | 32.60 | 32.60 | 160.21 | **0.0000** | **0.11** |
| conc:week | | | 9 | 0.13 | 0.01 | 4.29 | **0.0000** | **0.04** | 9 | | 23.83 | 2.65 | 13.01 | **0.0000** | **0.08** |
| conc:type | | | 3 | 0.01 | 0.00 | 0.54 | 0.6566 | 0.00 | 3 | | 19.65 | 6.55 | 32.20 | **0.0000** | **0.07** |
| week:type | | | 3 | 0.07 | 0.02 | 7.32 | **0.0001** | **0.03** | 3 | | 35.15 | 11.72 | 57.57 | **0.0000** | **0.12** |
| conc:week:type | | | 9 | 0.11 | 0.01 | 3.62 | **0.0002** | **0.03** | 9 | | 43.52 | 4.84 | 23.76 | **0.0000** | **0.14** |
| Residuals | | | 352 | 1.19 | 0.00 |  |  |  | 352 | | 71.63 | 0.20 |  |  |  |
| Amines | | | | | | | | | | | | | | | |
|  | | | Df | Sum Sq | Mean Sq | F value | Pr(>F) | ω^2^ | Df | | Sum Sq | Mean Sq | F value | Pr(>F) | ω^2^ |
| conc | | | 3 | 0.68 | 0.23 | 30.96 | **0.0000** | **0.13** | 3 | | 5.32 | 1.77 | 5.95 | **0.0006** | **0.02** |
| week | | | 3 | 1.11 | 0.37 | 50.93 | **0.0000** | **0.21** | 3 | | 49.16 | 16.39 | 54.93 | **0.0000** | **0.19** |
| type | | | 1 | 0.10 | 0.10 | 13.49 | **0.0003** | **0.02** | 1 | | 30.88 | 30.88 | 103.52 | **0.0000** | **0.12** |
| conc:week | | | 9 | 0.43 | 0.05 | 6.59 | **0.0000** | **0.07** | 9 | | 8.76 | 0.97 | 3.26 | **0.0008** | **0.02** |
| conc:type | | | 3 | 0.03 | 0.01 | 1.18 | 0.3185 | 0.00 | 3 | | 9.51 | 3.17 | 10.63 | **0.0000** | **0.03** |
| week:type | | | 3 | 0.13 | 0.04 | 5.83 | **0.0007** | **0.02** | 3 | | 27.93 | 9.31 | 31.20 | **0.0000** | **0.10** |
| conc:week:type | | | 9 | 0.15 | 0.02 | 2.25 | **0.0187** | **0.02** | 9 | | 20.88 | 2.32 | 7.78 | **0.0000** | **0.07** |
| Residuals | | | 352 | 2.57 | 0.01 |  |  |  | 352 | | 105.02 | 0.30 |  |  |  |
| Carboxylic Acids | | | | | | | | | | | | | | | |
|  | | | Df | Sum Sq | Mean Sq | F value | Pr(>F) | ω^2^ | Df | | Sum Sq | Mean Sq | F value | Pr(>F) | ω^2^ |
| conc | | | 3 | 0.15 | 0.05 | 32.58 | **0.0000** | **0.14** | 3 | | 1.18 | 0.39 | 13.17 | **0.0000** | **0.05** |
| week | | | 3 | 0.23 | 0.08 | 49.90 | **0.0000** | **0.21** | 3 | | 3.30 | 1.10 | 36.84 | **0.0000** | **0.15** |
| type | | | 1 | 0.02 | 0.02 | 12.18 | **0.0005** | **0.02** | 1 | | 2.58 | 2.58 | 86.48 | **0.0000** | **0.12** |
| conc:week | | | 9 | 0.06 | 0.01 | 4.40 | **0.0000** | **0.04** | 9 | | 0.47 | 0.05 | 1.74 | 0.0788 | 0.01 |
| conc:type | | | 3 | 0.01 | 0.00 | 1.70 | 0.1665 | 0.00 | 3 | | 0.38 | 0.13 | 4.20 | **0.0061** | **0.01** |
| week:type | | | 3 | 0.02 | 0.01 | 4.08 | **0.0072** | **0.01** | 3 | | 2.35 | 0.78 | 26.16 | **0.0000** | **0.11** |
| conc:week:type | | | 9 | 0.02 | 0.00 | 1.49 | 0.1509 | 0.01 | 9 | | 0.68 | 0.08 | 2.51 | **0.0085** | **0.02** |
| Residuals | | | 352 | 0.55 | 0.00 |  |  |  | 352 | | 10.52 | 0.03 |  |  |  |
| Variation | | | | | | | | | | | | | | | |
|  | | | Df | Sum Sq | Mean Sq | F value | Pr(>F) | ω^2^ | Df | | Sum Sq | Mean Sq | F value | Pr(>F) | ω^2^ |
| conc | | | 3 | 0.01 | 0.00 | 32.15 | **0.0000** | **0.14** | 3 | | 0.02 | 0.01 | 6.03 | **0.0005** | **0.02** |
| week | | | 3 | 0.01 | 0.00 | 26.88 | **0.0000** | **0.12** | 3 | | 0.13 | 0.04 | 42.91 | **0.0000** | **0.15** |
| type | | | 1 | 0.00 | 0.00 | 4.30 | **0.0388** | **0.01** | 1 | | 0.05 | 0.05 | 53.68 | **0.0000** | **0.06** |
| conc:week | | | 9 | 0.01 | 0.00 | 10.92 | **0.0000** | **0.13** | 9 | | 0.09 | 0.01 | 9.72 | **0.0000** | **0.09** |
| conc:type | | | 3 | 0.00 | 0.00 | 0.66 | 0.5780 | 0.00 | 3 | | 0.04 | 0.01 | 13.89 | **0.0000** | **0.05** |
| week:type | | | 3 | 0.00 | 0.00 | 2.75 | **0.0426** | **0.01** | 3 | | 0.06 | 0.02 | 21.60 | **0.0000** | **0.07** |
| conc:week:type | | | 9 | 0.00 | 0.00 | 2.29 | **0.0165** | **0.02** | 9 | | 0.11 | 0.01 | 12.18 | **0.0000** | **0.12** |
| Residuals | | | 352 | 0.05 | 0.00 |  |  |  | 352 | | 0.35 | 0.00 |  |  |  |
